# Head-neck movement may predispose to the development of arytenoid dislocation in the intubated patient: a 5-year retrospective single-center study

**DOI:** 10.1186/s12871-021-01419-1

**Published:** 2021-07-31

**Authors:** Eun-A Jang, Kyung Yeon Yoo, Seongheon Lee, Seung Won Song, Eugene Jung, Joungmin Kim, Hong-Beom Bae

**Affiliations:** Department of Anesthesiology and Pain Medicine, Chonnam National University Medical School, Chonnam National University Hospital, 160, Baekseo-ro, Dong-gu, Gwangju, 501 746 Korea

**Keywords:** Arytenoid dislocation, Head movements, Complication, Endotracheal intubation

## Abstract

**Background:**

Arytenoid dislocation is a rare laryngeal injury that may follow endotracheal intubation. We aimed to determine the incidence and risk factors for arytenoid dislocation after surgery under general anaesthesia.

**Methods:**

We reviewed the medical records of patients who underwent operation under general anaesthesia with endotracheal intubation from January 2014 to December 2018. Patients were divided into the non-dislocation and dislocation groups depending on the presence or absence of arytenoid dislocation. Patient, anaesthetic, and surgical factors associated with arytenoid dislocation were determined using Poisson regression analysis.

**Results:**

Among the 25,538 patients enrolled, 33 (0.13%) had arytenoid dislocation, with higher incidence after anterior neck and brain surgery. Patients in the dislocation group were younger (52.6 ± 14.4 *vs* 58.2 ± 14.2 yrs, *P* = 0.025), more likely to be female (78.8 *vs* 56.5%, *P* = 0.014), and more likely to be intubated by a first-year anaesthesia resident (33.3 *vs* 18.5%, *P* = 0.048) compared to those in the non-dislocation group. Patient positions during surgery were significantly different between the groups (*P* = 0.000). Multivariable Poisson regression identified head-neck positioning (incidence rate ratio [IRR], 3.10; 95% confidence interval [CI], 1.50–6.25, *P* = 0.002), endotracheal intubation by a first-year anaesthesia resident (IRR, 2.30; 95% CI, 1.07–4.64, *P* = 0.024), and female (IRR, 3.05; 95% CI, 1.38–7.73, *P* = 0.010) as risk factors for arytenoid dislocation.

**Conclusion:**

This study showed that the incidence of arytenoid dislocation was 0.13%, and that head-neck positioning during surgery, less anaesthetist experience, and female were significantly associated with arytenoid dislocation in patients who underwent surgeries under general anaesthesia with endotracheal intubation.

## Background

Endotracheal intubation during general anaesthesia can lead to complications such as submucosal hemorrhage, subglottic edema or laryngitis, vocal cord immobility, arytenoid dislocation and tracheal stenosis. Hoarseness, main symptom of these complications, has been reported with an incidence as high as 14.4% to 50% after general anaesthesia, although it is prolonged or permanent in 1% of patients who undergo surgery under general anaesthesia [1]. Among the complications, arytenoid dislocation (presenting as hoarseness, breathy voice, vocal fatigue, swallowing difficulty, and sore throat) is a very rare laryngeal injury, occurring in less than 0.1% of patients after general anaesthesia [2, 3]. In clinical practice, the symptoms of arytenoid dislocation are, therefore, sometimes overlooked as a possible cause of postoperative hoarseness and dysphagia. Moreover, arytenoid dislocation is easily misdiagnosed as vocal fold paralysis, because this dislocation alters normal laryngeal function and impairs airway protection as well [3, 4].

Hoarseness following endotracheal intubation is temporary and improves within several days in most patients. In patients with persistent hoarseness, arytenoid dislocation should be considered. When this complication is early diagnosed and promptly treated, the prognosis is generally favorable [5]. However, arytenoid dislocation can affect patient satisfaction and activities of daily living, even after discharge from the hospital [3]. Therefore, anaesthetists are very concerned about the occurrence of this event [6]. Moreover, a delay in diagnosis and treatment can lead to progressive fibrosis of the cricoarytenoid joint and subsequent vocal fold immobility. As such, identification of the risk factors for this complication may reduce its occurrence by enabling clinicians to avoid its triggers.

Because of the apparent rarity of arytenoid dislocation, it has primarily been described in case reports; systematic investigations have been rare [2, 7–14]. Several risk factors for this complication have been reported, including the use of a lighted stylet [2], laryngeal mask airway, or double-lumen tube [7]. Other factors include difficult intubation [2, 9, 12, 13], a cardiovascular operation [9], high body mass index [11, 13] and prolonged duration of operation [10, 14]. However, there has been few systemic study regarding clinical risk factors that can predict the occurrence of arytenoid dislocation. This retrospective study was, therefore, aimed to determine the incidence of, and the patient, and anaesthetic and operative factors associated with arytenoid dislocation in patients who underwent surgery under general anaesthesia with endotracheal intubation.

## Methods

This retrospective study protocol was approved (approval no.: CNUHH-2019–021) by the Institutional Review Board of Chonnam National University Hwasun Hospital (322, Seoyang-ro, Hwasun-eup, Hwasun-gun, Jeollanam-do, Republic of Korea), and was registered at the Clinical Research Information Service of the Korea National Institute of Health (trial no.: KCT0003640, 19/03/2019), which belongs to the World Health Organization Registry Network. The study protocol was performed in accordance with the Declaration of Helsinki and laws and regulations of the countries in which the clinical study was conducted, including data protection laws, the Clinical Investigation Agreement and the Clinical Investigation Plan. The requirement for written informed consent was waived by the review board because of the retrospective study design and lack of risk to patients. Data were manually retrieved and patients with a recorded diagnosis of arytenoid dislocation were identified retrospectively from the Chonnam National University Hwasun Hospital’s electronic medical record system. All available information about the patients was then entered into the study database using Microsoft Excel (Microsoft, NY, USA).

Patients 19-yr of age or older, who underwent surgery under general anaesthesia with endotracheal intubation from January 1, 2014 to December 31, 2018 were included. Patients were excluded from the analysis if they were younger than 18-yr of age, had undergone an emergency operation, tracheostomy, supraglottic airway device insertion, or double-lumen-endotracheal intubation. Patients were also excluded if their trachea was already intubated, or if they had any missing medical data needed for this study. Supraglottic airway devices, because they do not sit in the ideal position in the larynx [15], can also cause trauma to the airway. However, we excluded the patients with those devices insertion because the reported incidence is less than that caused by endotracheal tubes [16]. We also excluded the patients with double-lumen intubation because the size of double-lumen tube is much bigger than that of single one and thus the frequency of arytenoid dislocation may differ between the two tubes [7]. For all included patients with arytenoid dislocation, the occurrence of this complication had been confirmed by an otolaryngologist at the Department of Otorhinolaryngology-Head and Neck Surgery in our hospital, using a combination of fiberoptic laryngoscopy, computed tomography, and/or electromyography, at the time of consultation or referral, with postoperative hoarseness as the main symptom.

To identify risk factors for arytenoid dislocation, data on patient characteristics, anaesthetic factors, and surgical factors were collected. Patient characteristics included age, sex, body weight, height, body mass index, American Society of Anaesthesiologists physical status classification, and a short neck or limited mouth opening. Short neck and limited mouth opening are routinely assessed in our hospital; thus, this information is available in perioperative medical records. Limited mouth opening was defined as a mouth opening restriction of less than two finger breadths. Anaesthetic factors included Cormack grade, number of intubation attempts, size of endotracheal tube, the use of intubating tools, a stylet, an esophageal stethoscope, or the backward-upward-rightward pressure (BURP) maneuver, presence or absence of neuromuscular monitoring device, and degree of skills of anaesthetist (i.e., resident in year 1–4 of anaesthesia training, or an attending anaesthetist). Anaesthetists start to assess the degree of muscle paralysis immediately after induction of general anaesthesia, and intubate the patients about 90 s after administration of recuronium when train of four ratio reach zero. We routinely record the number of attempts at intubation, in the anaesthetic records.

Surgical factors included the position of intubated patients during surgery, especially in relation to head-neck movement (i.e., extension, flexion, or rotation). Other surgical factors included the duration of surgery and use of pneumoperitoneum. The position of the endotracheal tube has been reported to change significantly, with head-neck movement [17], as well as both with pneumoperitoneum alone and pneumoperitoneum with Trendelenburg positioning [18]. Meanwhile, movement of the tube and cuff in the trachea during surgery is known to increase the risk of postoperative throat complaints [19]. Thus, we determined whether the movement of the endotracheal tube is related to an injury to cricoarytenoid joint during the surgery. The primary outcomes were the incidence and risk factors for arytenoid dislocation after endotracheal intubation, with the aim to provide a basis for identification of high-risk patients and for further development and refinement of prediction models.

### Statistical analysis

Continuous data are presented as means ± standard deviation for normally distributed data and medians (interquartile range) for non-normally distributed data, and were compared using the unpaired Student’s t-test or Wilcoxon rank-sum test, as appropriate. The normality of the data was verified using the Shapiro–Wilk test. Categorical variables are presented as numbers (%), and were compared using Pearson’s χ^2^ or Fisher's exact test. Multivariable Poisson regression, which is suitable for modeling rare event data, was performed to determine the risk factors for arytenoid dislocation. First, univariable Poisson regression was performed to identify candidate variables (*P* < 0.2) for inclusion in the multivariable model. Variables were selected for forward and backward stepwise regression analyses based on the Akaike information criterion. Incidence rate ratios (IRRs) with 95% confidence intervals (CIs) were estimated according to the exponential of the regression coefficient for each variable. *P* < 0.05 was considered statistically significant; all tests were two-sided. The statistical analysis was performed using R software (version 3.6.0; R Foundation for Statistical Computing, Vienna, Austria).

## Results

**Fig. 1:**
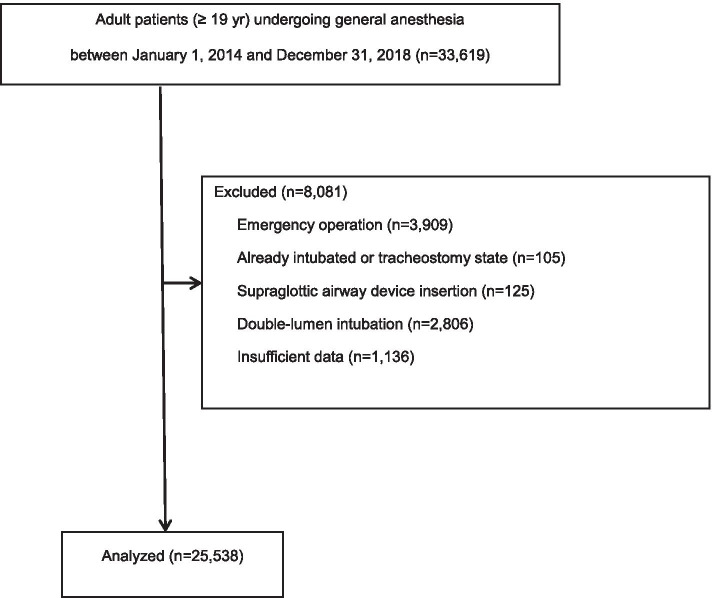
Patient screening and exclusion process

Of the 33,619 patients initially enrolled during the study period, 8,081 were excluded because they had undergone emergency operations (*n* = 3,909), were already intubated or had a tracheostomy (*n* = 105), underwent an operation using a supraglottic airway device (*n* = 125), underwent double-lumen intubation (*n* = 2,806), or had insufficient data (*n* = 1,136) (Fig. 1). The remaining 25,538 patients were included in the final analyses; of which 33 (26 women and 7 men; 0.12%) experienced arytenoid dislocation.

Demographic characteristics at baseline were comparable between the non-dislocation and dislocation groups, except that the patients in the latter group were younger (52.6 ± 14.4 *vs* 58.2 ± 14.2 yrs, *P* = 0.025) and more likely to be female (78.8 *vs* 56.5%, *P* = 0.014) (Table 1). Table 2 shows the anaesthesia-related characteristics: the incidence of intubation performed by a first-year anaesthesia resident was higher in the dislocation group than in the non-dislocation group (33.3 *vs* 18.5%, *P* = 0.048). In addition, positions during surgery were significantly different between the groups (*P* < 0.0001) (Table 3). Table 4 shows the results of univariable Poisson regression to determine potential risk factors for arytenoid dislocation. All variables with *P* < 0.2 in univariable regression were included in the multivariable Poisson regression analysis. In multivariate analysis, positions involving head-neck movement during surgery in intubated patients (IRR, 3.10; 95% CI, 1.50–6.25, *P* = 0.002), tracheal intubation by a first-year anaesthesia resident (IRR, 2.30; 95% CI, 1.07–4.64, *P* = 0.024) and female gender (IRR, 3.05; 95% CI, 1.38–7.73, *P* = 0.010) remained significant for increasing the risk of arytenoid dislocation (Table 5).

**Table 1:** Patient Characteristics

Variable	Non-dislocation (*n* = 25,505)		Dislocation (*n* = 33)		*P*-value
Age, yrs	58.2	± 14.2	52.6	± 14.4	0.025
Female, gender	14,288	(56.5%)	26	(78.8%)	0.014
ASA physical status					
I	6587	(25.8%)	14	(42.4%)	0.170
II	16,009	(62.8%)	17	(51.5%)	
III	2845	(11.2%)	2	(6.1%)	
IV	64	(0.3%)	0	(0%)	
Height, cm	161.1	± 8.6	160.5	± 9.0	0.663
Body weight, kg	62.4	± 11.5	62.7	± 12.3	0.914
Body mass index, kg m^−2^	24.0	± 3.6	24.2	± 3.4	0.781
Short neck	152	(0.6%)	0	(0%)	1.000
Mouth opening limitation	101	(0.4%)	0	(0%)	1.000

Values are presented as mean ± SD or number (%). *ASA* American Society of Anesthesiologists

**Table 2:** Anesthetic factors associated with arytenoid dislocation

Variable	Non-dislocation (*n* = 25,505)		Dislocation (*n* = 33)		*P*-value
Cormack Lehane grade					0.462
1	18,832	(73.8%)	28	(84.8%)	
2	5684	(22.3%)	5	(15.2%)	
3	973	(3.8%)	0	(0%)	
4	16	(0.1%)	0	(0%)	
Number of intubation attempts					0.999
1	24,794	(97.2%)	32	(97.0%)	
2	697	(2.7%)	1	(3.0%)	
3	13	(0.1%)	0	(0%)	
4	1	(0%)	0	(0%)	
Stylet use	939	(3.7%)	1	(3.0%)	1.000
BURP maneuver	2445	(9.6%)	1	(3.0%)	0.326
Tracheal intubation tool					
Conventional laryngoscope	25,077	(98.3%)	33	(100.0%)	0.967
Video-laryngoscope	327	(1.3%)	0	(0%)	
Lightwand	83	(0.3%)	0	(0%)	
Fiberoptic laryngoscope	18	(0%)	0	(0%)	
Endotracheal tube ballooning	25,413	(99.6%)	33	(100%)	1.000
Esophageal stethoscope	23,686	(92.9%)	30	(90.9%)	0.921
Neuromuscular monitoring	9531	(37.4%)	8	(24.2%)	0.169
Armoured tube	193	(0.8%)	1	(3.0%)	0.617
Endotracheal tube size (ID, mm)					0.237
< 6	14	(0%)	0	(0%)	
6	202	(0.8%)	1	(3.0%)	
6.5	260	(1.0%)	1	(3.0%)	
7	14,526	(57.0%)	26	(78.8%)	
7.5	66	(0.3%)	0	(0%)	
8	10,434	(40.9%)	5	(15.2%)	
8.5	2	(0%)	0	(0%)	
9	1	(0%)	0	(0%)	
Tracheal intubation by 1st-yr anaesthesia residents	4707	(18.5%)	11	(33.3%)	0.048

Data are presented as number (%). *ID* internal diameter, *BURP* backward upward rightward pressure

**Table 3:** Surgical factors associated with arytenoid dislocation

Variable	Non-dislocation (*n* = 25,505)		Dislocation (*n* = 33)		*P*-value
Duration of surgery, min	145.0	(105.0–210.0)	150.0	(105.0–255.0)	0.153
Pneumoperitoneum	8171	(32.0%)	6	(18.2%)	0.129
Patient position during surgery					0.000
Supine without neck movement	18,423	(72.2%)	15	(45.4%)	
Prone without neck movement	359	(1.4%)	0	(0%)	
Lateral without neck movement	1598	(6.3%)	1	(3.0%)	
Supine with neck extension	4621	(18.1%)	9	(27.3%)	
Supine with neck flexion	124	(0.5%)	0	(0%)	
Supine with neck rotation	280	(1.1%)	2	(6.0%)	
Lateral with neck flexion and rotation	100	(0.4%)	6	(18.1%)	

Data are presented as median (interquartile range) or number (%)

**Table 4:** Univariable Poisson regression of factors associated with arytenoid dislocation

Variable	IRR	95% CI	*P*-value
Patient characteristic			
Age	0.974	0.951–0.997	0.026
Gender			
Male	1.0		
Female	2.912	1.335–7.280	0.012
ASA physical status			
I	1.0		
II	0.500	0.247–1.031	0.055
III	0.331	0.052–1.185	0.144
IV	0.000	0.000–0.000	0.986
Height	0.991	0.952–1.032	0.664
Body weight	1.002	0.972–1.030	0.914
Body mass index	1.013	0.921–1.107	0.781
Short neck	0.000	–	0.987
Mouth opening limitation	0.000	–	0.984
Anaesthetic factor			
Cormack Lehane grade			
1	1.0	–	
2	0.592	–	0.280
3	0.000	–	0.986
4	0.000	–	0.998
Number of intubation attempts			
1	1.0	–	
2	1.111	–	0.917
3	0.000	–	0.987
4	0.000	–	0.996
Endotracheal tube ballooning	1.1525	0.046–3.796	0.984
Stylet use	0.818	0.017–1.370	0.843
BURP maneuver	0.295		0.229
Tracheal intubation tool			
Conventional laryngoscope	1.0		
Video–laryngoscope	0.000	–	0.987
Lightwand	0.000	–	0.998
Fiberoptic laryngoscopy	0.000	–	0.998
Esophageal stethoscope	0.768	0.274–3.205	0.663
Monitoring of neuromuscular blockade	0.537	0.227–1.139	0.126
Armoured tube	4.082	0.229–18.953	0.166
Tracheal intubation by 1^st^-yr anesthesia resident (*vs* higher than 1^st^ resident and staff)	2.206	1.030–4.454	0.032
Surgical factor			
Duration of surgery (min)			
< 120	1.0		
120–240	1.129	0.503–2.689	0.773
> 240	1.674	0.654–4.288	0.274
Pneumoperitoneum	0.472	0.176–1.067	0.096
^a^Positions with head-neck movement (*vs* positions without head-neck movement)	2.925	1.439–5.803	0.002

^a^Head-neck movement includes extension, flexion, rotation, and flexion-rotation. *ASA* American Society of Anesthesiologists, *BURP* backward upward rightward pressure, *IRR* incidence rate ratio, *CI* confidence interval

**Table 5:** Multivariable Poisson regression of factors associated with arytenoid dislocation

Variable	IRR	95% CI	*P*-value
Female gender (*vs.* male)	3.05	1.38–7.73	0.010
Tracheal intubation by 1^st^-yr anaesthesia residents (*vs* higher than 1^st^ resident and attending anaesthetist)	2.30	1.07–4.64	0.024
^a^Positions with head-neck movement (*vs* positions without head-neck movement)	3.10	1.50–6.25	0.002

^a^Head and neck movement includes extension, flexion, rotation and flexion-rotation. *IRR* incidence rate ratio, *CI* confidence interval

## Discussion

In a single-center retrospective study conducted over a 5-year period, 33 (0.12%) of 25,538 patients who underwent surgery under general anesthesia with endotracheal intubation experienced arytenoid dislocation. Head-neck positioning of intubated patients during surgery, less anesthesiologist experience, and female gender were significantly associated with increased incidence of arytenoid dislocation after general anesthesia.

The reported incidence of arytenoid dislocation varies widely among studies from 0.01% [11] to 0.1% [3, 19]. The incidence of this complication in our study (~ 0.13%) is consistent with rates (0.1%) reported by other researchers [3, 19]. Some patients with arytenoid dislocation might not have been referred to an otolaryngologist, instead recovering spontaneously without any manipulation. In addition, arytenoid dislocation is frequently misdiagnosed as recurrent laryngeal nerve paralysis [3, 4]. Moreover, the incidence of arytenoid dislocation may differ greatly among the type of surgery; it may be higher after bariatric/metabolic surgery with orogastric tube insertion (0.8%) [13], or in patients undergoing thyroid surgery (0.29%) as observed in the current study. These factors may explain why the incidence of arytenoid dislocation differs greatly among studies.

The mechanisms underlying arytenoid dislocation following intubation have not yet been determined, although the event is regarded as a type of intubation trauma. Paulsen et al. [20] attempted to replicate arytenoid dislocation in cadaveric larynges using tracheal intubation, extubation, and manual manipulation. However, the replication failed and it was thus concluded that arytenoid dislocation did not occur as a result of tracheal intubation alone. Moreover, Friedman et al. [21] evaluated the likelihood of arytenoid dislocation based on the force applied during tracheal intubation in cadaveric human larynges. However, they also failed to replicate arytenoid dislocation, even at maximum force, and concluded similarly that force applied during tracheal intubation was unlikely to cause this complication. These two studies raise questions regarding how arytenoid cartilage is dislocated due to intubation (or some other cause). Here, we evaluated demographic, anaesthetic, and surgical characteristics as potential risk factors for arytenoid dislocation.

Patients involving head-neck positioning during surgery had a significantly greater risk for arytenoid dislocation in the current study (IRR = 3.10, *P* = 0.002). It has been reported that the tip of the tube in the trachea is displaced up to a median of 5.0 cm (range: 3.5–7.0 cm) with head-neck movement [17], and that displacement of the tube in the trachea during surgery increases the risk of postoperative throat complaints [19, 22]. Moreover, anteromedial dislocation has been suggested to occur during intubation due to snagging of the arytenoid cartilage by the laryngoscope, tracheal tube, or stylet. Posterolateral dislocation has been proposed to occur during extubation with an incompletely deflated tracheal cuff [23]. Overall, it is suggested that up and down displacement of the cuffed tracheal tube, along with head positioning, may have caused inadvertent trauma to the cricoarytenoid joint, leading to arytenoid dislocation. Another explanation includes that the displaced cuffed tracheal tube or the convex curvature of the tracheal tube may have exerted prolonged pressure against the arytenoid cartilage and thereby inadvertently dislocated it during surgery. This speculation is supported by the findings that an endotracheal tube exerts pressure often in excess of 200 mmHg in the region of the arytenoid cartilage when the tube is left in situ in dogs [24], and that prolonged duration of anaesthesia is a significant risk factor for the occurrence of arytenoid dislocation [10, 14].

It is noteworthy that, as well as intubation/extubation itself, head-neck movement is causally related to arytenoid dislocation in intubated patients during surgery. To the best of our knowledge, this is the first report to suggest head-neck positioning as a risk factor for arytenoid dislocation during surgery. Indeed, head movement in tracheally intubated patients is a prerequisite for better surgical exposure in specific type of surgeries (e.g., anterior neck surgery or tracheal resection). In the current study, 17 (51.5%) of 33 patients experienced arytenoid dislocation while the neck was extended (*n* = 9; 27.3%), flexed with rotation (*n* = 6; 18.2%), or rotated (*n* = 2; 6.1%) during head and neck surgery. This may explain why arytenoid dislocation occurs in some patients despite uneventful endotracheal intubation under optimal intubation conditions [8]. Thus, it appears necessary to properly reassess the tube positioning, along with head-neck movement, and to avoid applying unnecessary pressure to the cuff by measuring the pressure immediately after intubation and regularly during prolonged intubation, or incomplete deflation thereof, before extubation.

Our study also demonstrated that intubation by a first-year anaesthesia resident was an independent risk factor for arytenoid dislocation, suggesting that the technical skills of the operator performing endotracheal intubation are important. This result is not surprising, because arytenoid dislocation is proposed to result from inadvertent trauma to the cricoarytenoid joint during insertion of airway tools into the larynx [25]. It has been reported that year of residency training is significantly associated with multiple tracheal intubation attempts leading to severe airway complications [26, 27]. In addition, considerable experience is required before a trainee becomes proficient in direct laryngoscopic tracheal intubation [28]. Thus, considerable operator experience and supervision by an attending anaesthetist [29] appear necessary to avoid arytenoid dislocation.

The finding that female patients (*vs* male patients) were almost three times as likely to develop arytenoid dislocation (IRR = 3.05, *P* = 0.010) is puzzling, because in a few previous studies gender was not associated with arytenoid dislocation [9, 11, 13] Postoperative sore throat and hoarseness have been reported to be more common in women, probably due to the smaller larynx and tighter endotracheal tube fitting compared to men [22, 30, 31]. In addition, female gender has been associated with a higher incidence of postoperative complications, such as sore throat, hoarseness, nausea, and vomiting, probably due to differences in anatomical structure, hormonal effects, or emotional expression [32]. Likewise, women are more likely to develop arytenoid dislocation after even minor intubation trauma. Another possible explanation for the gender difference may be type I error, which is more likely in smaller studies. Our university hospital has many thyroid surgeries, with the rate thereof being 4.9-fold higher in women than in men [33]; furthermore, head-neck extension is required for optimal surgical exposure. Notably, 9 (27.3%) of our 33 patients developed arytenoid dislocation after thyroid surgeries. Further studies are needed to confirm whether the gender difference was due to a reporting bias, or whether women are in fact at greater risk of arytenoid dislocation.

Although difficult intubation is considered a risk factor for arytenoid dislocation [2, 9, 12, 13], we found that Cormack grade, number of intubation attempts, or the use of an intubation stylet or BURP maneuver was not related to the occurrence of arytenoid dislocation. In addition, although a few studies have reported that body mass index [11, 13], use of a orogastric tube [14] or esophageal stethoscope, and a longer duration of surgery [10, 14] were risk factors for arytenoid dislocation, this was not the case in the current study. The discrepancies among studies are not readily explained. Previous studies were case reports [2, 7, 8] or compared patients with arytenoid dislocation to matched controls [10, 11]. The current study analyzed adults from the general patient population, all of whom underwent surgeries with endotracheal intubation, by using multivariable Poisson regression, which is suitable for modeling rare event data. It is likely that the low incidence rate of arytenoid dislocation and limited number of difficult intubation cases are responsible for the discrepancies among studies, which necessitates further studies with sufficient power. Alternatively, as Paulsen et al. [20] suggested, the occurrence of arytenoid dislocation is not related to tracheal intubation alone.

This study has several limitations. First, due to its retrospective design, it did not reflect differences in the subjective evaluations of anaesthetists who performed the intubations. For example, the Cormack grade, which is considered an objective airway assessment, might differ among examiners for a given patient [34]. Second, this study was performed at a single-centre, which limits the generalizability of the results. The findings should be confirmed by prospective, randomized, controlled, and sufficiently powered studies with larger patient populations or a multiple-centre design. Third, only patients with arytenoid dislocation who were referred to the Department of Otorhinolaryngology-Head and Neck Surgery of our hospital were included in the current study. Thus, the incidence of this complication may have been underestimated, because many patients may not have been consulted for treatment. Their symptoms may have resolved without treatment, or they may have visited other hospitals for treatment. Finally, arytenoid dislocation is known to arise from patient comorbidities, including laryngomalacia, renal insufficiency, acromegaly, and chronic steroid use. However, this study may have involved a selection bias for risk factors (e.g., comorbidities) due to its retrospective design based on analysis of electronic medical records, which prevented adjustment for other confounding factors.

## Conclusions

In conclusion, this study showed that arytenoid dislocation is a rare (but severe) complication, with an incidence of 0.13% after endotracheal intubation during general anaesthesia. We identified significant risk factors for arytenoid dislocation, including head-neck positioning in intubated patients during surgery, less anaesthetist experience, and female gender. Increased awareness of predictive factors could help to avoid arytenoid dislocation and improve patient outcome.

## Data Availability

The analyzed data sets generated during the study are available from the corresponding author on reasonable request.

## Abbreviations

BURP: Backward-upward-rightward pressure
IRRs: Incidence rate ratios
CIs: Confidence intervals
